# Human V6: Functional Characterisation and Localisation

**DOI:** 10.1371/journal.pone.0047685

**Published:** 2012-10-24

**Authors:** Velia Cardin, Rachael Sherrington, Lara Hemsworth, Andrew T. Smith

**Affiliations:** Psychology Department, Royal Holloway, University of London, Egham, Surrey, United Kingdom; University of Minnesota, United States of America

## Abstract

Human visual area V6, in the parieto-occipital sulcus, is thought to have an important role in the extraction of optic flow for the monitoring and guidance of self-motion (egomotion) because it responds differentially to egomotion-compatible optic flow when compared to: (a) coherent but egomotion-incompatible flow (Cardin & Smith, 2010), and (b) incoherent motion (Pitzalis et al., 2010). It is not clear, however, whether V6 responds more strongly to egomotion-incompatible global motion than to incoherent motion. This is relevant not only for determining the functional properties of V6, but also in order to choose optimal stimuli for localising V6 accurately with fMRI. Localisation with retinotopic mapping is difficult and there is a need for a simple, reliable method. We conducted an event-related 3T fMRI experiment in which participants viewed a display of dots which either: a) followed a time-varying optic flow trajectory in a single, egomotion-compatible (EC) display; b) formed an egomotion-incompatible (EI) 3×3 array of optic flow patches; or c) moved randomly (RM). Results from V6 show an ordering of response magnitudes: EC > EI > RM. Neighbouring areas V3A and V7 responded more strongly to EC than to RM, but about equally to EC and EI. Our results suggest that although V6 may have a general role in the extraction of global motion, in clear contrast to neighbouring motion areas it is especially concerned with encoding EC stimuli. They suggest two strategies for localising V6: (1) contrasting EC and EI; or (2) contrasting EC and RM, which is more sensitive but carries a risk of including voxels from neighbouring regions that also show a EC > RM preference.

## Introduction

The visual system integrates local motion signals across the retina for two distinct purposes. The first is to detect and characterise the motion of large objects in the visual scene. Such objects may be rigid, in which case common local motion vectors are identified and unified, but they may also be non-rigid (e.g. a flock of birds), in which case the local motion signals may be quite disparate (individual paths of birds) but nonetheless yield a strong sense of overall global motion. The second purpose is to extract visual information that can be used to detect and monitor self-motion or “egomotion”. Movement of the head generates characteristic retinal distributions of image velocity known as optic flow and these can be detected and used to specify self-motion.

Human V6 is a visual area in the dorsal part of the parieto-occipital sulcus (POS). fMRI studies have shown that this region responds differentially to optic flow patterns when compared to random motion [1], and also when compared to coherent global motion that is not consistent with self-motion and must therefore reflect object motion [2]. The latter finding suggests the specialisation of this area in the processing of stimuli that are relevant for self-motion, rather than global object motion. V6 also responds more strongly to optic flow patterns when they are combined with coherent disparity gradients which could contribute information about distance and position of objects in space [3], and to object motion, but not retinal motion, during pursuit, in particular if combined with self-motion compatible flow fields [4]. Furthermore, neurons in the homologous area in macaque respond selectively to “real motion”, have large receptive fields (up to 80 deg) and, unusually, their responses represent peripheral and foveal regions equally [5], [6]. Taken together, these results highlight the role of V6 in extracting visual cues for the representation of self-motion, as opposed to global motion of objects.

In humans, V6 is located in the dorsal portion of the parieto-occipital sulcus (POS) [7]. The location of this hemifield map is somewhat variable (see [2] for a detailed description): in some cases it is dorsal to V2, and abutting V3 and V3A, whereas in other cases it is located in a more dorsal/anterior position, closer to V7 than V3. Given that human V6 is a relatively small visual area, and (as in macaques) does not seem to have a preferential foveal representation [7], [8], [9], localisation of V6 with retinotopic mapping is laborious, difficult and sometimes unreliable. It requires a wide-field stimulus for good results and can fail completely with stimuli of the size typically available in scanners. Therefore development of a simple, reliable method for the localisation of V6 in neuroimaging studies is needed, particularly in view of growing evidence for its central role in representing self-motion and the consequent need for further study of V6. As mentioned above, V6 shows a differential response to egomotion-compatible optic flow both when compared to random motion and when compared to egomotion-incompatible optic flow. Given that both contrasts result in a significant activation of V6, it has been suggested in both cases that they can be used to localise V6. However, partly because the functional properties of area V6 are not fully understood, it is not known if both methods identify this area with the same efficacy, and even though both studies suggest that the activations obtained with the respective contrasts overlap with V6 as identified retinotopically, a direct comparison that allows determination of whether both identify the same functional area has not been made.

Human V6 has been studied much less than most other visual areas, such as V1–V4, MT+ and LOC. The aim of this study is to characterise further the functional responses of human V6 and, in doing so, to provide guidelines for a reliable and quick method for its localisation that will be helpful as the future study of V6 develops. For this purpose, human V6 was first identified retinotopically using fMRI, after which its responses to EC, EI and random motion were determined. The extent of activation elicited by these conditions was also evaluated in areas adjacent to V6, not only to determine the functional response of these areas to different types of visual motion but also because significant activation of these areas by a V6 localiser could potentially contaminate the definition of V6.

## Materials and Methods

### Main Experiment

#### Ethics statement

The study was conducted in accordance with the Declaration of Helsinki and approved by the local ethics committee at Royal Holloway, University of London. Standard MRI screening procedures were followed for all participants, who gave their written consent to participate in the study and were paid for their participation.

#### Participants

7 individuals (5 women; age 18–56) with normal or corrected-to-normal vision took part in the study.

#### Stimuli

The stimuli were generated using OpenGL libraries in C++ and projected onto a screen in the bore of the scanner using a LCD projector. To obtain wide-field visual stimulation, emulating natural optic flow, the screen was viewed via a custom optical device that magnified the image. The device was monocular and was positioned over the participant’s preferred eye; the unstimulated eye was occluded.

**Figure 1:**
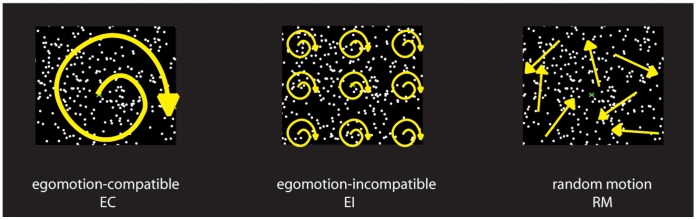
Stimuli. The stimuli consisted of random dot kinematograms. There were three conditions: 1) egomotion-compatible (EC), with dots expanding, contracting and rotating from the centre of the display; 2) egomotion-incompatible (EI), with dots expanding, contracting and rotating from nine equi-distant points in the display; and 3) random motion (RM), consisting of dots moving in random directions.

The stimuli consisted of 800 moving dots arranged in an egomotion-consistent (EC), egomotion–inconsistent (EI), or random motion (RM) pattern (see Fig. 1). The first two conditions were very similar to the stimuli used in our previous work [2], [10]. The EC condition consisted of a 42°×42° square field of dots moving in a coherent optic-flow pattern containing expansion/contraction and rotation components that varied over time, consistent with self-motion on a varying spiral trajectory [11], displayed at 60 fps. For a given dot with radius *r*, angle θ and local speed *v*, its trajectory was defined by:
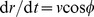


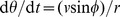



Radial and angular velocities are defined by d*r*/d*t* and dθ/d*t,* respectively. The direction of optic flow was defined by φ, which varied over time from −π to π generating a stimulus with radial, circular and spiral motion. The local speed did not vary with distance from the origin to avoid local speed confounds between the EC and EI stimuli (pilot fMRI results show that responses are similar for (i) stimuli with constant speed and size and (ii) stimuli with radially increasing speed and size).

The use of time-varying flow ensured that all locations were stimulated by all dot directions during the course of the stimulus cycle. It gives larger responses than (say) continuous expansion, perhaps because multiple flow-sensitive neurons are stimulated. It also ensures that adaptation at any one local direction is minimal. The EI stimulus consisted of a 3×3 array of nine identical panels, each containing a smaller version of the EC stimulus. Although the individual panels contain optic flow, the overall pattern is not consistent with egomotion because flow induced by observer motion can have only one centre of motion. In the RM condition, the dots moved in independent random directions. Each dot moved in a straight path, and when it disappeared from the display, it was randomly repositioned and given a new direction. In order to equate low-level visual characteristics, the dot size, dot speed and number of dots in the whole array were kept identical across conditions. In addition, by the nature of the stimuli, all local directions were presented (at different times) at all points in the image in all three conditions, so there were no differences between conditions in time-averaged local direction.

Each stimulus was presented for 3 s in an event-related design, with inter-trial intervals (ITI) in which the screen was blank (apart from a central fixation spot). The ITIs varied between 2 s and 10 s, following a near-Poisson probability distribution (mean ITI = 5.62 s). A scanning session consisted of six experimental runs, the order counterbalanced across participants. Each run had 30 trials (10 per condition) presented in a pseudo-random order, plus a 10 s buffer at the beginning and the end, and lasted in total 4 min 48 s. Participants were instructed to fixate a small central square that changed colour throughout the run at a rate of 2.5 Hz. To ensure fixation and to minimise fluctuations in attention, participants performed a task that consisted of counting the number of instances of a particular colour.

#### Data acquisition

Images were acquired with a 3T MR scanner (Magnetom Trio, Siemens, Erlangen, Germany) equipped with a custom 8-channel posterior-head array headcoil (Stark Contrast, Erlangen, Germany). Functional images were acquired with a standard gradient-echo, echoplanar sequence (repetition time [TR] = 2500 ms, echotime [TE] = 31 ms, flip angle = 85°, voxel size = 3×3×3 mm, 35 axial slices, bandwidth = 1410 Hz/pixel). For coregistration purposes, at the beginning of each scanning session, we also acquired two single-volume echo-planar imaging (EPI) sequences that had the same position parameters as the experimental runs: one (BC-EPI) using the scanner’s integral whole-body coil to give uniform contrast and another immediately after, acquired with the posterior array head coil (PA-EPI). In the same scanning session, an anatomical T1-weighted image was acquired (MPRAGE, 160 axial slices, in-plane resolution 256×256, 1 mm isotropic voxels, TR = 1830 ms, TE = 4.43 ms, flip angle = 11°, bandwidth = 130 Hz/pixel).

In a different session, a high-resolution T1-weighted 3D anatomical image [modified driven-equilibrium Fourier transform, MDEFT; [12], 176 axial slices, in-plane resolution 256×256, 1 mm isotropic voxels, TR = 7.92 ms, TE = 2.45 ms, flip angle = 16, bandwidth = 195 Hz/pixel] was acquired using a standard (whole-head) Siemens 8-channel array head coil. MDEFT was chosen in place of standard 3D anatomical sequences because of its improved contrast between gray matter and white matter, which is beneficial for segmentation and flattening. This anatomical image was used as a reference to which all the functional images were co-registered.

#### Data analysis

All data were pre-processed and analyzed with BrainVoyager QX (version 2.0, Brain Innovation, The Netherlands). EPIs were corrected for head motion and slice timing. To remove low frequency drifts, a general linear model (GLM) with 2 pairs of sine/cosine predictors of 1 and 2 cycles per run, and a linear regressor, was used to estimate the low frequency components of the timeseries and subtract them from the original data. No spatial smoothing was applied. All functional images were aligned to the PA-EPI acquired at the beginning of the scan session. Due to the steep posterior-to-anterior intensity gradient of the EPIs acquired with the posterior array head coil, direct coregistration of these images to the anatomy was found to be poor. Therefore, we coregistered the BC-EPI to the MPRAGE structural image corrected for inhomogeneities, and assumed no head movements between the acquisition of the BC-EPI and the PA-EPI. The MPRAGE was also coregistered to the MDEFT image. Coregistration accuracy was always checked visually. Parameters from both coregistration steps were then used to build functional 3-D timeseries in the same space as the MDEFT image.

Analysis was conducted by fitting a GLM, with regressors representing the three stimulus categories and six movement parameters. For every experimental condition, each stimulus presentation was modelled as a boxcar of 3 s duration, convolved with a canonical haemodynamic response function (HRF), and entered into a multiple regression analysis to generate parameter estimates for each regressor at every voxel. Movement parameters were derived from the realignment of the images and included in the model. The first three volumes of each run were discarded to allow for T1 equilibration effects. Correction for effects of serial autocorrelations was applied using the first order autoregression AR(1) method.

Percent signal changes for each experimental condition were extracted from each independently defined region of interest (see following section) and averaged across hemispheres. For each participant, results were normalised by dividing values for all conditions within a single ROI by the maximum value for that ROI. For visualisation of the activations elicited by the comparison of the experimental conditions, appropriate contrasts were defined individually for each participant and the results overlaid on each person’s MDEFT or its flattened representation.

### Retinotopic Mapping

#### Participants, stimuli and data acquisition

The same 7 individuals participated. Wide-field retinotopic mapping was performed to demarcate areas V1d, V2d, V3d, V3A, V7 and, where possible, V6. Polar angle and eccentricity were mapped using standard retinotopic mapping procedures [13], [14]. For polar angle mapping, a counterphasing checkerboard “wedge” stimulus (a 24° sector of approximately 30° radius) rotated clockwise at a rate of 64 s/cycle (six cycles per run). A counterphasing ring was used for eccentricity mapping; this ring increased in radius until reaching 30° eccentricity, also at a rate of 64 s/cycle (six cycles per run). The duty cycle varied between 5% (fovea) to 25% (periphery). Check size was scaled by eccentricity in approximate accordance with the cortical magnification factor. Three wedge and three eccentricity stimulus runs were performed in a separate scanning session from the main experiment. Stimuli were projected in the same way as in the main experiment, and presented monocularly with the same optical device. Images were acquired and pre-processed as in the main experiment, but in this case volumes consisted of 28 slices and TR = 2000 ms.

#### Data analysis

Data were analyzed by fitting a model to the timecourse obtained with the retinotopic mapping stimulation. This consisted of a rectangular wave of duty cycle 24/360, reflecting the duration of stimulation at any portion of the visual field, convolved with the HRF. The phase of the fitted response was taken as an index of visual-field location, in terms of polar angle or eccentricity. A flattened representation of each hemisphere was created by segmenting and reconstructing the border between grey and white matter within each hemisphere of the MDEFT scan using BrainVoyager 2.0. The resulting surfaces were smoothed, inflated, and cut along the calcarine sulcus. Finally, the surface was flattened and corrected for linear distortions. Reversals of the direction of phase change across the cortical surface in the polar angle map were taken as boundaries of visual areas. The boundaries of visual areas V1–V7 were drawn by eye, on the basis of these reversals. V6 was defined with reference to the description provided by Pitzalis et al. (2006) [7]; we looked for a complete hemifield representation and/or an independent eccentricity map, located close to the peripheral visual field representations of V2d, V3d and V3A. In hemispheres in which V6 could be identified by both an eccentricity and a polar angle map, only those voxels that were part of both maps were included in the ROI definition.

## Results

### Functional Response of Area V6 to Visual Motion

**Figure 2:**
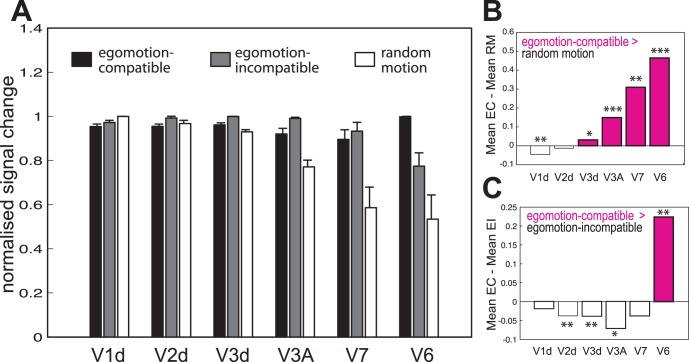
ROI analysis. **A)** For each hemisphere, the average percent signal change was obtained across all the voxels in each retinotopically defined ROI. The bars represent the mean normalised signal change across hemispheres, ±1 S.E.M., for each of the experimental conditions. **B and C)** Response obtained by subtracting the mean response in RM (**B**) and EI (**C**) from the mean EC response. Positive values represent a higher response for the EC condition. *p<0.05; **p<0.005; ***p<0.001.

Wide-field dot-kinematograms were used to characterise the functional response of V6 and surrounding areas. Three conditions were tested: random motion (RM), egomotion-incompatible optic flow (EI) and egomotion-compatible optic flow (EC). V6 was localised using large field retinotopic mapping (see Methods). For comparison, the responses of neighbouring visual areas V1d, V2d, V3d, V3A and V7 were also evaluated. The objectives of these comparisons were to distinguish V6 functionally from other surrounding areas that also respond well to visual motion stimulation, and to determine to what extent contrasts between different conditions activate V6 exclusively. V1d, V2d, V3d, V3A and V7 were localised in all the tested hemispheres (14/14). V6, which is much harder to localise with retinotopic mapping, was identified in 11/14 hemispheres. To avoid biases in the results due to potential individual differences, analysis of the imaging data from V1-V3A and V7 only included ROIs from those hemispheres where V6 was also indentified. Percent signal changes for each experimental condition were extracted from each ROI of each hemisphere, and averaged across participants. Fig. 2A shows the normalised response of all ROIs to all the experimental conditions (See Fig. S1 for results without normalisation). Areas V3d (t_(10)_ = 2.47,p = 0.002), V3A (t_(10)_ = 6.75, p = 0.00005), V7 (t_(10)_ = 3.82, p = 0.0033) and V6 (t_(10)_ = 4.41, p = 0.0013) all show a significantly higher response to EC than to RM (Fig. 2A, B). However, V6 (t_(10)_ = 3.85, p = 0.003) is the only area that responds significantly more to EC than to EI. This is very clear in Fig. 2C, which plots the difference between the two conditions. Areas V3d (t_(10)_ = 7.3, p = 0.00003), V3A (t_(10)_ = 7.5, p = 0.00002), V7 (t_(10)_ = 4.01, p = 0.0025) and V6 (t_(10)_ = 3.85, p = 0.0032) also show a significantly higher response to EI than to RM (Fig. 2A).

### Functional Localisation of V6

**Figure 3:**
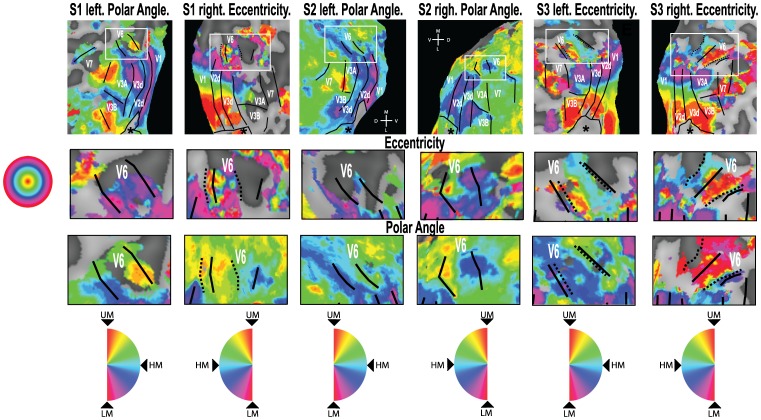
Retinotopic maps for 3 participants. Retinotopic maps overlaid on flattened representations of the dorsal portion of the occipital lobe. **Top row:** For each participant, both hemispheres are shown and, in each case, either a polar angle map or an eccentricity map is overlaid onto the dorsal part of the occipital lobe. The continuous black lines show the border of each retinotopic area as defined by the polar angle maps. It was not possible to determine the upper vertical meridian border of V3B in S2 left. The dashed black line shows the definition of area V6 based on the eccentricity maps. The white frame shows the region enlarged in the middle and bottom rows. The approximate location of the foveal confluence is indicated with a star (*). M – medial; L – lateral; D – dorsal; V – ventral. Middle and Bottom row: Eccentricity maps and polar angle maps, respectively, of the V6 area. The colour wheels indicate position in the visual field represented by each colour in the retinotopic maps of polar angle and eccentricity. UM – upper vertical meridian; LM – lower vertical meridian; HM – horizontal meridian.

**Figure 4:**
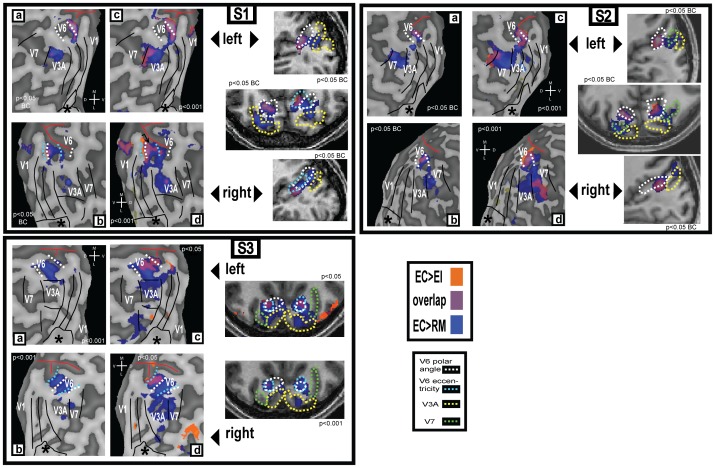
Activation maps for 3 participants. (**a–d**): The same flattened occipital representations from Fig 3, overlaid with the activations obtained with the two functional contrasts (see colour key top right). Activations are shown thresholded at two different levels in each case. corr: Bonferroni corrected. The continuous black lines show the borders of retinotopic areas V1–V7 as defined by the polar angle maps. The dashed white and cyan lines show the definitions of area V6 based on the polar angle and eccentricity maps, respectively. The red line indicates the position of the parieto-occipital sulcus. The approximate location of the foveal confluence is indicated with a star (*). M – medial; L – lateral; D – dorsal; V – ventral. (**right hand column**): Activations overlaid onto slices from each participant’s structural scan. In the axial slices, the right hemisphere is shown on the right.

Due to its small size and lack of cortical magnification, it is challenging to localise V6 using retinotopic mapping. A large visual stimulus is required [7], which is usually not practical without special equipment, and even then maps of V6 are sometimes indistinct and occasionally absent. Maps for both hemispheres in each of three participants are shown in Fig. 3 and Fig. S2. The full map shown for each hemisphere in Fig. 3 is either a polar angle map or an eccentricity map, depending on which map showed V6 more clearly. The remaining maps are shown in Fig. S2. However, an amplified image of the V6 region is shown both for the polar angle and the eccentricity map for each participant. We found it easier to define V6 based on the polar angle data than on the eccentricity data - V6 could be defined on the polar angle map in all six hemispheres shown in Fig. 3, but an eccentricity map can only be observed in the right hemisphere of S1 and both hemispheres of S3. In some cases, the definition based on polar angle coincides perfectly with the eccentricity definition (Fig. 3, S3 left), but in other cases it differs (Fig. 3, S1 right). As an alternative method for localising V6 that is both more practical and more reliable, two previous studies have suggested the use of contrasts between the motion conditions tested in this study: [EI > RM] [1], and [EC > EI] [2]. Both contrasts revealed significant differential activation of V6. Fig. 4 (a–d) shows the results of these statistical contrasts overlaid on the same flattened representations of the occipital lobes of the same participants for whom retinotopic maps are shown in Fig. 3. They are also shown on slices from the anatomical scans (Fig. 3, right hand side figures on each subject box). Each panel identifies, in different colours (see key), regions that are responsive to each of the two contrasts. These results are in agreement with the ROI results presented in Fig. 2, which show that both comparisons result in significant differential activity in V6. Overall, they demonstrate not only that both comparisons can be used to localise this area, but also that both localise broadly the same functional area.

However, there are some important differences between the two contrasts. Comparing Figs 2 A and C, it is clear that the comparison [EC > EI] is the only one that causes a significant activation in V6 and not in the neighbouring areas. Furthermore, when results for this contrast, shown in orange in Fig. 4, are overlaid onto the flatmaps and anatomical slices, it is possible to define V6 easily by delineating the area of activation in the POS. In contrast, the activation obtained with the contrast [EC > RM], shown in blue in Fig. 4, tends not to be confined to V6 but also to extend into neighbouring areas V3A and V7, as would be expected given that both these areas also show a differential response for this contrast in the ROI analysis (Fig. 2 A and B). In some cases, when overlaid onto a flatmap (e.g. Fig. 4, S1a and S3b), the additional activity is not contiguous with that in V6 and does not compromise localisation. However, when this activation is overlaid at the same conservative threshold onto the anatomical scan (e.g. Fig. 4, S1 middle right), it fills a continuous region encompassing both V6 and parts of V3A/V7, making identification of the boundaries of V6 impossible. It should be noticed that the differential activation in V3A/V7 is sometimes observed at conservative significance thresholds (e.g. p<0.05 corrected for multiple comparisons; Fig. 4, S1 and S2 left and right hand panels), whereas the activation obtained with the contrast [EC > EI] does not spread into neighbouring areas at such thresholds. V6 could still be defined well with the contrast [EC > RM] in these cases with the additional use of retinotopic maps to identify the neighbouring areas (Fig. 3), and because some of the activations are clearly not located in the POS (e.g. Fig. 4, S2 top right corner). However, it would be difficult to define V6 reliably simply by overlaying the results of this contrast on an anatomical scan without knowing where the other retinotopic areas are located.

As noted above, the problem varies considerably among participants. Although in many cases the contrast [EC > EI] is more effective than [EC > RM] in isolating V6, this is not always the case. In the example shown in Fig. 3, S3a, the contrast [EC > RM] does not spread into other areas but activates only the region in the POS corresponding to V6. Moreover, the activation with this contrast is considerably more robust and extensive than that resulting from the [EC > EI] contrast. Although the use of [EC > RM] risks including parts of neighbouring areas in the region demarcated as V6, the significance and magnitude of the response to this contrast is always greater than that obtained with [EC > EI]. This greater sensitivity, which is expected in light of Fig. 2, could make [EC > RM] the more satisfactory comparison of the two where sensitivity is limited, for example if there are constraints on the amount of scanning time that can be allocated to the localiser scans.

For reference, we have included a map of the activations obtained with the contrast [EI > RM] (Fig. S3). As expected from the results presented in Fig. 2, this contrast results in widespread activation of retinotopic dorsal areas, including V6, which does not make it ideally suited for localisation of this area. But more importantly, the aim of localising V6 with these contrasts is to isolate a region that is selective for visual motion that is consistent with self-motion – this criterion will not be satisfied if the localisation contrast does not include an egomotion-compatible condition.

## Discussion

In this study we have shown that human V6 responds well to visual motion, with response magnitudes ordered EC > EI > RM. These results emphasise the selectivity of this area for egomotion-compatible (EC) optic flow and its importance in the extraction of visual cues to self-motion. They also show a hierarchical increase in selectivity for coherent motion in the dorsal visual cortex, where V3A and V7 respond preferentially to coherent motion but do so whether the likely origin is self-motion or object-motion, whereas V6 responds selectively to EC optic flow. Our results also demonstrate that the two functional contrasts used by different research groups for the localisation of V6, [EI > RM] [1], and [EC > EI] [2], result in activations that overlap in the POS. Even though localisation of V6 using retinotopic mapping is challenging, the accumulated retinotopic maps of V6 from this study and previous ones [1], [2], [7], show that the activations obtained using contrasts between motion stimuli to localise this area overlap well with its retinotopic definition.

The area labelled V6, in this and previous studies, is thought to be homologous to macaque V6. However, in macaque, area V6 is adjacent to V6A [15], the exact location of which has not been identified in the human brain. In macaque, V6A is heavily involved in the control of reaching and grasping and has more limited visual sensitivity than V6 [16], [17], [18], [19]. For this reason, it seems unlikely that the region identified here with retinotopic mapping and functional localisers corresponds primarily to a V6A homologue, but it is not possible to be definite about this without a clear definition of V6A in the human brain. Since macaque V6A has many visually responsive cells and these are thought to inherit some response properties from V6, it could be possible that some voxels that correspond to V6A are included in the V6 ROIs defined here. However, we think it likely that macaque V6A (or possibly one of its dorsal and ventral subdivisions; [20]) is homologous with human SPOC (superior parieto-occipital cortex) as identified with reaching and grasping tasks [21], [22]. Whereas SPOC appears to be located in the POS but close to the dorsal surface of the brain, human V6 is typically located a little more ventrally in the POS and, in our view, is more likely to be adjacent to SPOC than coincident with it. The dorsal position of SPOC and its involvement in reaching are both consistent with a V6A homology. If this analysis is correct, it suggests that the V6 region we discuss in this paper is indeed only V6 and does not include V6A.

**Figure 5:**
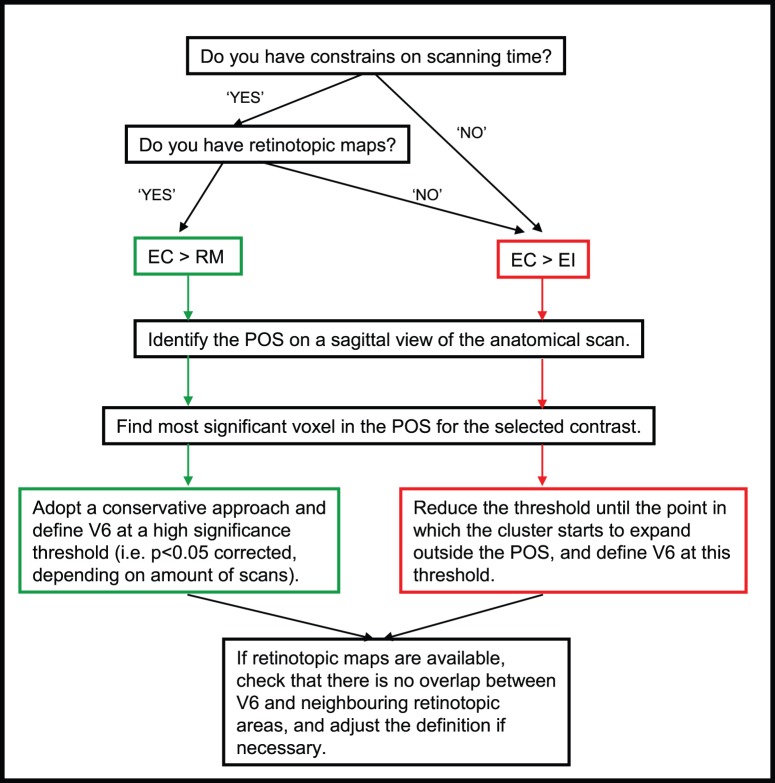
Guidelines for the functional localisation of human V6. EC: egomotion-compatible; EI: egomotion-incompatible; RM: random motion.

### Differences in Localisation between Methods

Although our results confirm that both contrasts activate V6, they also show there are some important differences between them in the extent of the activation obtained. The contrast [EC > RM] results in strong activation of V6, but it also tends to activate neighbouring retinotopic areas (V3d, and in particular V3A and V7), which can potentially contaminate the definition of V6 with voxels from other functional regions. This could be problematic if V6 is simply defined as all voxels that are significantly active with the contrast [EC > RM]. The spread to neighbouring retinotopic regions is made less problematic if the V6 definition is manually edited, for example to exclude voxels outside the POS, but this requires the use of subjective criteria. Similarly, applying a high significance threshold can assist, but this could result in a conservative definition of the area that includes only a subset of the voxels that truly correspond to V6.

On the other hand, the contrast [EC > EI] results in a cluster of activity in the POS that is more reliable in the sense that voxels outside V6 are unlikely to be erroneously included. However, because the difference between EC and EI is smaller than that between EC and RM (Fig. 2), this contrast is considerably less sensitive. Consequently longer scanning sessions may be required in order to obtain significant activations, which is problematic if scanning-time constraints exist.

The stimuli in this study differ somewhat from the stimuli used in Pitzalis et al 2010 [1] – they use a 16s block design, with a jittered centre of flow and varied speed. It is not possible for us to make recommendations with regard of the best type of stimuli and experimental design without a systematic comparison of each of the parameters. However, both studies use time-varying optic flow, which is likely to be ideal because it stimulates neurons with different flow sensitivities, and it reduces the adaptation at any one local direction. In terms of the monocular stimulation used in this study, it was only done for the purpose of obtaining a wide field of view. Even though V6 is sensitive to coherent disparity gradients [3], and disparity cannot play a role with only one stimulated eye, binocular stimulation without coherent disparity fields has been used successfully before for the localisation of V6 (see Supplementary Fig. 2 in [2], and [1]).

It is difficult to localise human V6 using retinotopic mapping because this is a relatively small cortical region, with no foveal magnification. The difficulty is not surprising if we take into account that (i) traditional retinotopic mapping techniques stimulate different discrete regions of the visual field at different times, which reduces considerably the total activity obtained from a particular voxel, and (ii) the stimuli used consist of alternating high-contrast checkerboards, which are unlikely to drive V6 strongly. For localisation of V6 with retinotopic mapping, it is indispensable to use wide-field stimulation, to at least stimulate as much of the area as possible. However, this is not the case with the contrasts mentioned in this study. Even though we use wide-field stimulation here, we have shown before [2] that a standard field size (around 20 deg), results in good localisation of area V6 with the contrast [EC>EI]. We have no data with smaller fields for the contrast [EC>RM], but given that the difference between these conditions in V6 is even larger for this contrast, it is likely that small fields will also work well with this comparison. This is not the case with standard field retinotopic mapping – in our 2010 study [2], we could only localise V6 in 5 of 22 hemispheres retinotopically mapped with a 12 deg radius wedge.

In this study, we identified V6 retinotopically in 11/14 hemispheres either using polar angle or eccentricity data. In the remaining three hemispheres, a definition of V6 was not possible without the aid of the activations obtained with the [EC>EI] and [EC>RM] comparisons, and therefore we decided to exclude these three hemispheres from our analysis.

### Functional Definition of V6: Recommendations

Taking into account all the issues discussed above, we propose some simple guidelines for the definition of V6 using functional contrasts (Fig. 5). If there are no scanning constraints or if retinotopic maps are unavailable, we recommend the use of the contrast [EC > EI], given that this contrast is more likely to activate V6 exclusively. However, if the scanning time constraints are important and if retinotopic data are available, the [EC > RM] contrast would be a better option.

In both cases, we recommend a flexible approach to thresholding, because using a fixed statistical threshold leads to very variable definitions across individuals. For example, in some participants, it is possible to isolate V6 well with the contrast [EC > EI], but at a very low significance threshold (see Fig. 4, S3). In view of cases like this, a fixed threshold is not practical. In our lab, we start by identifying the most significant voxel in the POS. We then reduce the significance threshold slowly until the cluster starts to spread outside the POS and we define V6 with a threshold just above this point. We do not suggest that V6 has to occupy the full width of the POS. However, the areas that are more likely to be contaminating V6 are V3A and V7, which are not located in the POS. Therefore, if a voxel positive for the contrasts discussed is in the POS, it is likely to correspond to area V6. The threshold of the contrast is not an issue if the definition of V6 is made aided by retinotopic definition of the surrounding areas, but it is particularly important if retinotopic definitions of the areas surrounding V6 are not available. In particular, it is important if researchers decide to use the contrast [EC > RM] without these maps. If this is the case, we recommend using a high significance threshold, such as p<0.05 (corrected for multiple comparisons). This a conservative approach and some V6 voxels may be excluded, but it will ensure that voxels included in the definition of V6 are part of this area, and not part of neighbouring areas.

### Conclusion

In summary, we have confirmed that human V6 has a preference for egomotion-compatible (EC) motion stimuli [2], but show that it nonetheless responds more strongly to egomotion-incompatible coherent motion (EI) than to random motion (RM), giving an ordering of response magnitudes: EC > EI > RM. Neighbouring areas V3A and V7 also respond more strongly to EC than to RM, but about equally to EC and EI. Based on these results, we suggest two main strategies for localising V6 with functional contrasts: (1) contrasting EC and EI, where extensive scanning is possible and a definitive result is required; or (2) for a faster, more sensitive but less conservative result, contrasting EC and RM, with the aid of standard retinotopic techniques to assist in exclusion of voxels from neighbouring regions that also show a EC > RM preference.

## Supporting Information

Figure S1 **ROI analysis without normalisation.** For each hemisphere, the average percent signal change was obtained across all the voxels in each retinotopically defined ROI. The bars represent the mean percent signal change across hemispheres, ±1 S.E.M., for each of the experimental conditions. The plot only show significant positive differences for the contrast [EC>EI] and [EC>RM] (*p<0.05; **p<0.01; ***p<0.001). There were only positive significant differences (at least p<0.05) for the contrast [EI>RM] in areas V3d, V3A, V7 and V6.
(EPS)

Figure S2 **Additional retinotopic maps for hemispheres shown in** **Figure 3** **.** Retinotopic maps overlaid on flattened representations of the dorsal portion of the occipital lobe. For each participant, both hemispheres are shown and, in each case, either a polar angle map or an eccentricity map is overlaid onto the dorsal part of the occipital lobe, complementing the maps shown in Fig. 3. The definitions of V7 and V3B in the right hemisphere of S3 are based on data from a single-run map, where the limits of these regions are clearer. The continuous black lines show the border of each retinotopic area as defined by the polar angle maps. The dashed black line shows the definition of area V6 based on the eccentricity maps. The white frame shows the region enlarged in the middle and bottom rows of Fig. 3. The approximate location of the foveal confluence is indicated with a star (*). M – medial; L – lateral; D – dorsal; V – ventral. For each hemisphere, the average percent
(TIF)

Figure S3 **Map of activations obtained with the contrast [EI>RM].** Activations of the contrast [EI > RM] overlaid on flattened representation of the dorsal occipital lobe. The continuous black lines show the border of each retinotopic areas defined by the polar angle maps. The dashed black line show the definition of area V6 based on eccentricity maps. The approximate location of the foveal confluence is indicated with a star (*). M – medial; L – lateral; D – dorsal; V – ventral.
(EPS)
