# Supplementary figures and images for: Human V6: Functional Characterisation and Localisation
Source: PLoS One. 2012 Oct 24;7(10):e47685. doi: 10.1371/journal.pone.0047685 (PMC3480433; doi:10.1371/journal.pone.0047685)

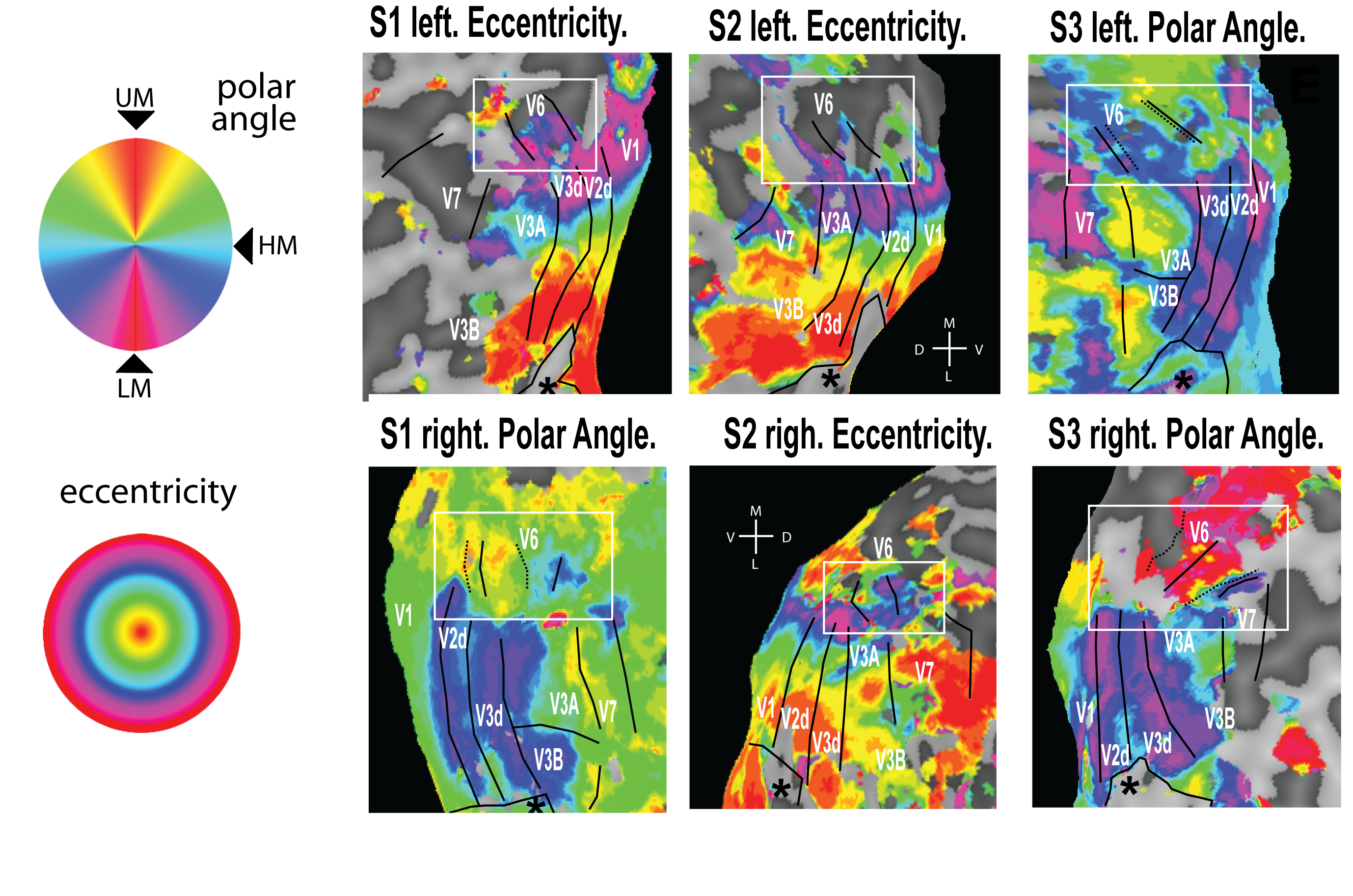

Supplement: Figure S2 — Additional retinotopic maps for hemispheres shown in Figure 3 . Retinotopic maps overlaid on flattened representations of the dorsal portion of the occipital lobe. For each participant, both hemispheres are shown and, in each case, either a polar angle map or an eccentricity map is overlaid onto the dorsal part of the occipital lobe, complementing the maps shown in Fig. 3. The definitions of V7 and V3B in the right hemisphere of S3 are based on data from a single-run map, where the limits of these regions are clearer. The continuous black lines show the border of each retinotopic area as defined by the polar angle maps. The dashed black line shows the definition of area V6 based on the eccentricity maps. The white frame shows the region enlarged in the middle and bottom rows of Fig. 3. The approximate location of the foveal confluence is indicated with a star (*). M – medial; L – lateral; D – dorsal; V – ventral. For each hemisphere, the average percent (TIF) [file pone.0047685.s002.tif]
